# Complete Genome Sequence of a Novel Reassortant H3N6 Avian Influenza Virus Isolated from Domestic Green-Winged Teal

**DOI:** 10.1128/genomeA.00250-13

**Published:** 2013-05-16

**Authors:** Chaochao Xiong, Qian Liu, Quanjiao Chen, Yanfeng Yao, Huadong Wang, Jianjun Chen

**Affiliations:** Center for Emerging Infectious Diseases, Wuhan Institute of Virology, Chinese Academy of Sciences, Wuhan, Hubei, China

## Abstract

An avian influenza virus strain, A/domestic green-winged teal/Hunan/2036/2007(H3N6) (DGW-T2036), was isolated from healthy domestic green-winged teals (*Anas crecca*) in Hunan Province, South China. All eight gene segments of the isolate were sequenced. Genomic analysis demonstrated that this H3N6 virus is a novel reassortant avian influenza virus with a gene constellation originating from multiple ancestors.

## GENOME ANNOUNCEMENT

Avian influenza virus, known as bird flu, is a member of the family *Orthomyxoviridae* and is classified into 16 hemagglutinin (HA) and 9 neuraminidase (NA) subtypes (1). It is well known that wild ducks are the major natural reservoir for low-pathogenicity avian influenza (LPAI) viruses and have a higher prevalence of influenza A virus than other species (2). In a previous study, we isolated two strains of H5N1 viruses, which were closely related to the viruses circulating in chickens and ducks, from apparently healthy domestic green-winged teals (3). These results suggested that domestic green-winged teals, a type of wild duck bred for meat, may play an important role in transmitting avian influenza virus.

In 2007, we collected cloacal specimens from domestic green-winged teals and isolated an avian influenza virus (AIV) H3N6 strain, which was designated A/domestic green-winged teal/Hunan/2036/2007 (DGW-T2036). We performed reverse transcriptase (RT)-PCR using universal primers for influenza A virus and sequenced the entire viral genome (4). The complete genome of this H3N6 strain consists of eight single-stranded RNA segments, PB2, PB1, PA, HA, NP, NA, M, and NS, with nucleotide lengths of 2,341, 2,341, 2,233, 1,765, 1,565, 1,419, 1,027, and 890, respectively.

Nucleotide homology comparisons revealed that the HA gene sequence of the DGW-T2306 virus is highly similar to that of A/duck/Beijing/40/04(H3N8), sharing 98% nucleotide identity, while its NA gene sequence is highly similar to that of A/duck/Eastern China/01/2007(H4N6). The NP gene sequence shares greatest identity (98%) with that of A/duck/Zambia/04/2008(H3N8). PB1 and PB2 gene fragments are most closely related to those of H3N8 isolate A/duck/Beijing/40/04 and H4N6 isolate A/wild duck/Korea/SH5-60/2008, sharing 99% and 98% nucleotide identity, respectively. PA and M genes are most similar to those of H6N8 isolate A/duck/Guizhou/1560/2007 (>99% identity) and H4N1 isolate A/swine/Hubei/06/2009(H4N1) (99% identity), respectively. The NS gene sequence exhibits the closest relationship (about 99% identity) to that of the H6N5 isolate A/aquatic bird/Korea/CN5/2009. These results indicate that the DGW-T2306 virus is a novel reassortant H3N6 virus derived from multiple ancestors.

Based on the deduced amino acid sequence for the HA gene, DGW-T2036 possesses residues GlnSerGly at positions 226 to 228, indicating an α2,3-linked sialic acid preference (3). No amino acid mutation was observed at His274Tyr in the NA protein, which indicates that DGW-T2036 should be sensitive to NA inhibitors. The PB2 segment of this virus possesses 158Glu, 627Glu, and 701Asp; PB1 has 436Tyr, and PA has 515Thr, suggesting low pathogenicity of this virus in mammals (5–7). The amino acids of the M2 protein transmembrane domain, such as 26Leu, 27Val, 30Ala, 31Ser, 34Gly, 37His, and 41Trp, present no mutations associated with drug resistance, revealing that the virus is sensitive to M2 inhibitors (8).

In summary, the H3N6 virus is a novel reassortant avian influenza virus with its gene constellation derived from multiple ancestors. These results highlight the importance of surveillance at live poultry markets.

### Nucleotide sequence accession numbers.

The genome sequence of A/domestic green-winged teal/Hunan/2036/2007(H3N6) has been deposited in GenBank under accession numbers KC709820 to KC709827.
